# Leukotriene receptor expression in esophageal squamous cell cancer and non-transformed esophageal epithelium: a matched case control study

**DOI:** 10.1186/s12876-016-0499-z

**Published:** 2016-07-30

**Authors:** M. Venerito, C. Helmke, D. Jechorek, T. Wex, R. Rosania, K. Antweiler, J. Weigt, P. Malfertheiner

**Affiliations:** 1 Department of Gastroenterology, Hepatology and Infectious Diseases, Otto-von-Guericke University Hospital, Leipziger Str. 44, 39120 Magdeburg, Germany; 2 Institute of Pathology, Otto-von-Guericke University Hospital, Leipziger Str. 44, 39120 Magdeburg, Germany; 3 Department of Biometrics and Medical Informatics, Otto-von-Guericke University Hospital, Leipziger Str. 44, 39120 Magdeburg, Germany

**Keywords:** Esophageal squamous cell cancer, Leukotriene receptors, Eicosanoids, Carcinogenesis

## Abstract

**Background:**

Leukotriene B4 (LTB4R and LTB4R2) and cysteinyl leukotriene receptors (CYSLTR1 and CYSLTR2) contribute to malignant cell transformation. We aimed to investigate the expression of LTB4R, LTB4R2, CYSLTR1 and CYSLTR2 in esophageal squamous cell carcinoma and adjacent non-transformed squamous epithelium of the esophagus, as well as in control biopsy samples from esophageal squamous epithelium of patients with functional dyspepsia.

**Methods:**

Expression of LTB4R, LTB4R2, CYSLTR1 and CYSLTR2 was analyzed by immunohistochemistry (IHC) and quantitative reverse transcription-polymerase chain reaction (qRT-PCR) in biopsy samples of 19 patients with esophageal squamous cell cancer and 9 sex- and age-matched patients with functional dyspepsia.

**Results:**

LTB4R, LTB4R2, CYSLTR1 and CYSLTR2 were expressed in all biopsy samples. Major findings were: 1) protein levels of all leukotriene receptors were significantly increased in esophageal squamous cell cancer compared to control mucosa (*p* < 0.05); 2) *CYSLTR1* and *CYSLTR2* gene expression was decreased in cancer tissue compared to control at 0.26–fold and 0.23–fold respectively; 3) an up-regulation of LTB4R (mRNA and protein expression) and a down-regulation of *CYSLTR2* (mRNA expression) in non-transformed epithelium of cancer patients compared to control (*p* < 0.05) was observed.

**Conclusions:**

The expression of leukotriene receptors was deregulated in esophageal squamous cell cancer. Up-regulation of *LTB4R* and down-regulation of *CYSLTR2* gene expression may occur already in normal squamous esophageal epithelium of patients with esophageal cancer suggesting a potential role of these receptors in early steps of esophageal carcinogenesis. Larger studies are warranted to confirm these observations.

**Electronic supplementary material:**

The online version of this article (doi:10.1186/s12876-016-0499-z) contains supplementary material, which is available to authorized users.

## Background

The incidence of squamous cell carcinoma of the esophagus (ESCC) varies widely in the world, with age-adjusted incidence rates ranging from 5 to 100/100.000 inhabitants/year [1]. In Europe, the incidence of this subtype of esophageal cancer is of 5.4/100.000 for men and 1.1/100.000 for women, with highest rates in Scotland (13/100.000 for men and 4/100.000 for women) and lowest rates in Greece and Bulgaria (below 2/100.000 for men and 0.5/100.000 for women) [2]. In Germany the incidence is of 6–10/100.000, occurring four times more often in men than in women [3]. The prognosis is poor, with only 1 on 5 patients surviving 3 years or more after the initial diagnosis [4]. Many epidemiological studies have demonstrated that hazardous alcohol consumption and tobacco smoking increase the risk of ESCC [5–7].

Leukotrienes belong to the large group of eicosanoids that originate from the oxidative degradation of arachidonic acids [8]. Eicosanoids have pleiotropic effects on various cellular functions and numerous studies have shown their role in the pathogenesis of chronic inflammation and cancer [9, 10]. In particular, recent studies have shown an involvement of leukotriene receptors in the development of carcinomas of the pancreas, stomach, colon, urinary bladder and ovary [11–15].

We hypothesized that the expression of these receptors might be deregulated also in ESCC. Therefore, the expression pattern of the two leukotriene B4 receptors LTB4R and LTB4R2 and the two receptors for cysteinyl leukotrienes (CYSLTR1 and CYSLTR2) was studied by immunohistochemistry and quantitative reverse transcription-polymerase chain reaction (qRT-PCR) in a prospective study cohort of patients with esophageal squamous cell cancer. Tissue specimen from cancer and adjacent non-transformed squamous epithelium were analyzed. Gene expression may also be deregulated in adjacent non-transformed squamous epithelium of patients with esophageal squamous cell cancer [16]. Thus, a control group of patients with functional dyspepsia was recruited.

## Methods

### Study population

The study was conducted according to the declaration of Helsinki of 1975, as revised in 1983 and was approved by the Ethics Committee of the Otto-von-Guericke University Hospital of Magdeburg (No. 34/08). All subjects provided written informed consent before entering the study. Nineteen newly diagnosed patients with ESCC were prospectively enrolled from March 2009 to April 2010 at the Otto-von-Guericke University Hospital, Magdeburg, Germany. After overnight fasting all individuals underwent upper gastrointestinal endoscopy with videogastroscope (GIF Q145 or GIF Q180, Olympus Medical, Hamburg, Germany). The following exclusion criteria were applied: malignancies other than ESCC, lack of signed informed consent, clinically instable patient. None of the patients with ESCC had chemotherapy, radiotherapy or surgery prior to endoscopy. For each patient four biopsies each were collected from the tumor and from macroscopically non transformed mucosa. Two biopsies were sent to the pathologist for histology, one biopsy was immediately snap-frozen in liquid nitrogen and stored at -80 °C and one other was directly paraffin embedded. Narrow-band imaging was used to better demarcate neoplastic lesions from the surrounding normal non neoplastic mucosa. Lugol’s chromoendoscopy was used to further investigate flat lesions suspicious of being cancerous.

Nine sex- and age-matched subjects (±4 years) undergoing upper GI endoscopy for dyspeptic symptoms were enrolled as control group. Exclusion criteria were: the presence of typical symptoms for gastro-esophageal reflux disease, the intake of proton pump inhibitors in the last 2 weeks, and/or the presence of esophageal erosions at endoscopy. Control biopsies were collected at least 2 cm cranial to the Z-line and subsequently snap frozen or directly paraffin embedded as mentioned above.

### Structured questionnaire

Patients enrolled prospectively and controls were interviewed within 2 days before undergoing upper GI endoscopy using a structured questionnaire, providing information on demographics, medical history, smoking habits, alcohol intake and proton pump inhibitor (PPI) intake. The english version of the study questionnaire is shown in the Additional file 1. As there is no safe level of smoking [17], patients were classified into current smokers (within the past 12 months), former smokers and patients who never smoked. Alcohol consumption was consequently classified in 3 categories as most guidelines in different countries recommend that alcohol intake should not exceed 20g/day for men and 10g/day for women [18]: abstainers, low alcohol consumption (<20g/day for men, <10g/day for women) and hazardous alcohol consumption (≥20g/day for men, ≥10g/day women).

### Extraction of total RNA, cDNA synthesis and quantitative RT-PCR

Biopsies were stored at -80 °C and subjected to a two-step RNA extraction protocol as described previously [19]. cDNA transcription was performed using 250 ng of total RNA amount. In a final volume of 40 μl, 20 units of AMV reverse transcriptase (Promega, Mannheim, Germany) in the buffer containing 1x reaction buffer, 0.5 mM dNTP (Roche, Mannheim, Germany), 10 mM random hexanucleotides and 50 units of placenta RNase inhibitor (all reagents from Promega) were utilized. After incubation at 42 °C for 1 h enzymes were inactivated at 95 °C for 10 min and the reaction mixture was kept frozen at -80 °C until enzymatic amplification. Quantitative RT-PCR was performed using an iCycler (BioRad, Munich, Germany). A typical 30 μl reaction mixture consisted of 15μl HotStarTaq™ Master Mix, 1.2 μl of the RT-reaction, 0.3 μl SYBR-Green I (1:10.000) (Molecular Probes, Eugene, USA), and 0.25 μM of the specific primers for the gene analyzed. Primary denaturation and activation of Taq-polymerase at 95 °C for 15 min was followed by 40 cycles with denaturation at 94 °C for 30 s, annealing at 60 °C for 30 s, and elongation at 72 °C for 30 s. The initial template mRNA amounts were calculated by determining the time point at which the linear increase of sample PCR product started, relative to the corresponding points of a standard curve; these are given as artificial units. All PCR products were cloned into the pDIRECT™ (Qiagen, Hilden, Germany) and used as internal standard for PCR. All PCR standard curves had correlation coefficients >0.95. β-actin mRNA amounts were used to normalize the cDNA contents of the different samples. The following primers were used for the RT-PCR analysis: *β-actin* (fw: 5’-cat-gcc-atc-ctg-cgt-ctg.gac-c-3’, rev: 5’-aca-tgg-tgg-tgc-cgc-cag-aca-g-3’), *LTB4R* (fw: 5′-tca-gca-cca-tca-ggg-cag-tga-c-3′, rev: 5′-ctg-acc-ctg-gga-ttg-gca-tca-g-3′), *LTB4R2* (fw: 5′-ggg-tgt-aaa-ggg-acg-tgc-aca-g-3′, rev: 5′-gct-tgt-gct-gtt-tcc-tgg-caa-g-3′), *CYSLTR1* (fw: 5′-caa-tag-tgt-cat-ggc-atg-tgg-c-3′, rev: 5′-gct-tgc-ttc-tga-gaa-caa-acg-c-3′), *CYSLTR2* (fw: 5′-AGG-ATT-GAA-GCA-GGC-ATT-GGC-3′, rev: 5′-aaa-gtg-gag-gtc-cca-gaa-tcg-g-3′).

### Immunohistochemical staining and cell count

**Fig. 1:**
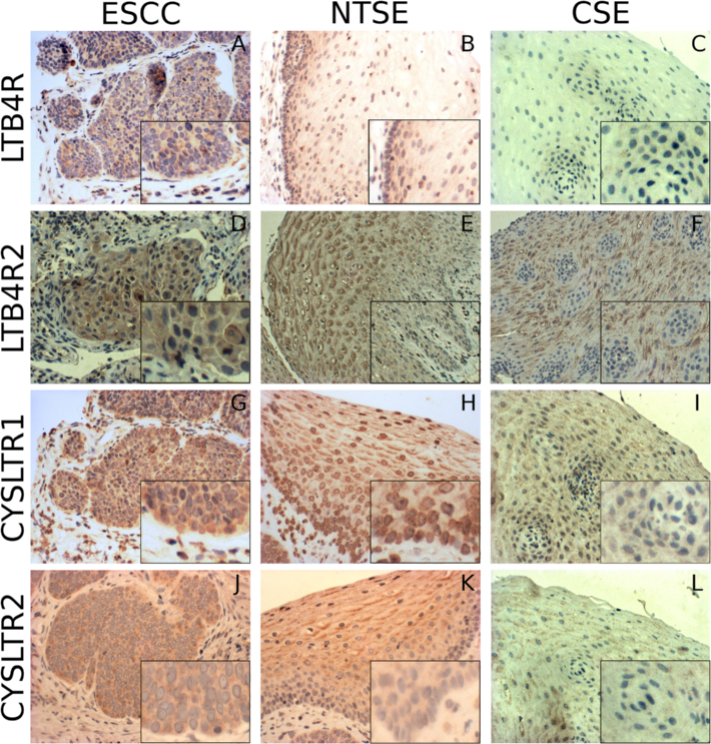
Immunohistochemical localization of LTB4R/LTB4R2 and CYSLTR1/2. Leukotriene receptors are horizontally displayed from the top to the bottom. Vertical sections represent the distinct histological tissues examined. Images show sections with low (*large* picture) and higher (*right lower* corner) magnification example. In non-transformed mucosa of cancer patients and control mucosa, details display a representative section from basal stratum with adjacent submucosal tissue. Furthermore, control panels show a mucosal papilla with circularly oriented basal membrane. Positive receptor detection appears as brown staining (microscope: Nikon F200 camera 990). For LTB4R (**a**-**c**), the receptor is predominately located within the cytosol of cancer cells and normal esophageal epithelium. Within the non-transformed epithelia LTB4R reaches medium (**b**) to low (**c**) staining intensity in basal cells and a further reduction in luminal areas. The reactions for LTB4R2 also show a cytosolic receptor pattern (**d**-**f**). Non-cancerous tissues (E and F) present low LTB4R2 expression in basal strata. An up-regulation of LTB4R2 can be seen in superficial epithelial layers instead. CYSLTR1 results are demonstrated in the pictures below (**g**-**i**). Cancerous and non-cancerous epithelial esophageal cells present a cytosolic staining. As depicted, CYSLTR1 is also located within the nuclei of cancer cells and non-transformed mucosa of patients with cancer (**g** and **h**). A weak CYSLTR1 expression is present in basal stratum of the normal esophageal epithelium of cancer patients with up-regulation in superficial layers. CYSLTR1 staining remains low across all cellular layers of dyspeptic control. CYSLTR2 is also localized within the cytoplasm (**j**-**l**). Both groups with normal esophageal epithelium display a weak CYSLTR2 reactivity in basal compartments, whereas CYSLTR2 is up-regulated in luminal areas (**i** and **l**). ESCC = esophageal squamous cell cancer; NTSE = non-transformed squamous epithelium of cancer patients; CSE = control squamous epithelium

Immunohistochemical analysis was performed using the avidin-biotin complex immunostaining method and the automated immunohistochemistry slide staining system by Ventana NexES (Ventana Medical System, Strasbourg, France). Tissue sections were deparafinized, dehydrated and underwent antigen retrieval using a Dako protocol. Slides were incubated with specific primary rabbit polyclonal antibodies for LTB4R, LTB4R2, CYSLTR1 or CYSLTR2 (Cayman chemicals, catalogue number 120114, dilution 1:100; Acris, catalogue number SP4368P, dilution 1:25; GeneTex Inc., catalogue number GTX70519, dilution 1:100; Lifespan Biosciences, catalogue number LS-A2255, dilution 1:100, respectively) either. All primary antibody incubations were followed by PBS-washing. Positive immunohistochemical reactions were revealed using the iVIEW^TM^ DAB Detection Kit (Ventana, Germany). Specimens were counterstained with hematoxylin and mounted with DEPEX™. Specificity of immunostaining was checked with non-immune serum. Samples were examined independently by CH and DJ. For LTB4R, LTB4R2, CYSLTR1 and CYSLTR2, the staining intensity (SI) and the percentage of positive cells (PP) were scored as followed: SI was classified in 0 (no staining), 1 (weak), 2 (moderate) and 3 (strong); PP: 0 (no positive cells), 1 (<10 %), 2 (10–50 %), 3 (51–80 %), 4 (>80 %). For each slide the immunoreactive score (IRS) was calculated as SI x PP with a possible maximum score of 12. The esophageal mucosa consists of different layers including (from the basal membrane to the lumen) basal stratum, spinosum stratum and superficial stratum. An example for an absent staining is shown in superficial cells of Fig. 1c (IRS = 0), whereas basal stratum shows a weak SI (SI score = 1) for 10–50 % of cells in the same picture (IRS = 2). A moderate staining is displayed in figure 1G (SI score = 2) for >80 % of cancer cells (IRS = 8). In 1H, superficial stratum shows a strong SI (SI score = 3) for >80 % cells (PP score = 4) resulting in an IRS of 12. For IRS assessment and statistical analysis we focused on the sole basal stratum where (cancer) stem cells are supposed to originate from [20].

### Statistical analysis

All data were entered into a database and analyzed using the R - 2.15.0. statistic software (free download on http://www.r-project.org/). Wilcoxon signed-rank test and Mann-Whitney *U* test were used for comparisons of the groups where appropriate (2-sided). A statistical *p*-value <0.05 was considered as significant for all comparisons. In order to reduce the chances of obtaining false-positive results due to multiple comparisons (*k* = 12), Bonferroni correction was applied to the *p*-value of comparisons concerning immunohistochemistry of ESCC and basal stratum of non-transformed epithelium in cancer patients and control (*p* < 0.05).

## Results

### Overview on demographic population characteristics and tumor location, staging and grading

Baseline data of ESCC patients are presented in Table 1. The majority of subjects with ESCC and functional dyspepsia were male (14/19 and 5/9 respectively), and the mean age was 62 ± 11 years in cancer patients and 61.6 ± 12 years in controls. The most common symptoms reported at admission from patients with ESCC were loss of weight (60 %) and dysphagia (74.7 %), whereas only 28 % of patients reported heartburn and 25.3 % odynophagia.

**Table 1:** Baseline data of ESCC patients

	*N* = 19 (%)
Male/female	14/5 (73.6/26.4)
Mean age (years)	62 ± 11
Alcohol consumption	
Abstainer	1 (5.3)
Former drinking	6 (31.6)
Active drinking	12 (63.2)
Alcohol consumption (amount)	
Low^a^	5 (41.7)
High^b^	7 (58.3)
Smoking habits	
Never	0
Former smoker	8 (42.1)
Active smoker	11 (57.9)

Subjects were considered former drinkers of alcohol and former smokers when no consumption was declared within the past 12 month, respectively. The amount of male and female alcohol intake was differently classified into (^a^) low (<20g/day for men, <10g/day for women) and (^b^) high risk (≥20g/day for men, ≥10g/day women) intake. *ESCC* esophageal squamous cell cancer

For each patient the alcohol consumption was also recorded. In 94.7 % of cases (18 of 19) alcohol consume was accounted; 12/19 patients (63.2 %) were active alcohol consumer and 6/19 cases (31.6 %) were former drinker. 1 patient (5.3 %) never drank alcohol. According to the amount of alcohol, patients were considered low alcohol consumer (5/19, 41.7%) and high alcohol consumer (7/19, 58.3 %). Also smoking habits were recorded and 11/19 cases (57.9 %) were considered active smoker, whereas 8/19 patients (42.1 %) were former smoker in the last year. There was no never smoker in cancer patients.

Control group was composed by 9 sex-age matched patients with dyspeptic symptoms: 5 men (55.5 %) and 4 female (44.5 %). For 6/9 controls, smoking habits and alcohol intake were recorded: 3/ 6 controls had never smoked and further 3 controls were former smokers. Four out of 6 controls reported to consume alcohol currently whereas 2 controls were abstainers. In 3 cases questionnaires were not filled out before endoscopy. The use of sedation for endoscopy hampered to obtain missing information afterwards. Patients were not contacted again. No data on quantity of alcohol was available for controls.

**Fig. 2:**
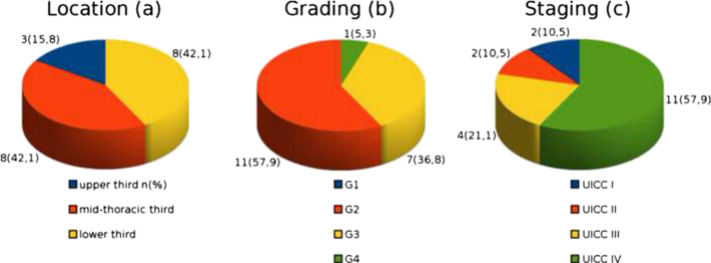
Tumor characteristics. An overview of (**a**) tumor location, (**b**) tumor grading and (**c**) staging is given from the *left* to the *right*. In (**a**), the tumor location is calculated as distance from *upper* central incisor teeth. *Upper*, mid-thoracic and *lower* third of the esophagus refer to 15 – 23 cm, 23 – 32 cm and 32 - 40 cm from *upper* incisor teeth, respectively. No G1 cancer was detected (**b**). Most cancers were in stage IV on initial presentation (**c**). *n* = absolute number of patients; UICC = Union Contre le Cancer

In Fig. 2 an overview on tumor characteristics is presented. Tumor location was documented as distance from upper central incisor teeth. Distribution on upper (15 – 23 cm), mid-thoracic (23 – 32 cm) and lower esophagus (32 – 40 cm) was 3 (15,8 %), 8 (42,1 %) and 8 (42,1 %).

With respect to the tumor grading, 0, 11/19 (57.9 %), 7/19 (36.8 %) and 1 (5.3 %) patients had G1, G2, G3 and G4, respectively.

According to the classification of the international Union Contre le Cancer [21] stage I, II, III and IV were present in 2/19 (10.5 %), 2/19 (10.5 %), 4/19 (21.1%) and 11/19 (57.9 %) patients, respectively. None of the patients in the control group had a malignancy of the upper GI tract.

### Immunohistochemical expression of leukotriene receptors in ESCC, non-transformed epithelium of cancer patients and controls

Immunohistochemical staining was performed for all cancers (19/19) and control patients (9/9), as well as 15/19 normal esophageal epithelia of cancer patients. 4/19 biopsies from normal esophageal epithelia of cancer patients contained insufficient tissue for immunohistochemical evaluation. Raw data on immunohistochemical expression of leukotriene receptors are provided in the Additional file 2. For each receptor staining, comparisons were performed between cancer tissue and basal strata of esophageal epithelium in cancer patients and control. An overview on LTB4R, LTB4R2, CYSLTR1 and CYSLTR2 receptors and their distinct expression profiles in cancers and non-malignant tissue is presented in Fig. 1. In cancerous and non-cancerous epithelial cells, leukotriene receptors were located within the cytoplasm. For CYSLTR1, nuclear receptor staining was also detected in 14/19 ESCC (74 %) as well as in 12/15 non-transformed mucosa specimen of patients with cancer (80 %) and 6/9 in control (66 %). In the present study we focused on the expression of leukotriene receptors in cancer tissue and basal epithelial cells of the esophageal mucosa. Statistical results regarding staining intensities of the different leukotriene receptors are shown in Table 2. IRS of LTB4R was medium in cancer tissue and basal stratum of esophageal epithelium in cancer patients, whereas in control epithelium basal cells presented a weak reactivity. LTB4R protein expression was increased in cancer and non-transformed epithelium of cancer patients compared to control (*p* < 0.05), whereas no difference in LTB4R expression was observed between ESCC and adjacent non-transformed epithelium in cancer patients. IRS of LTB4R2, CYSLTR1 and CYSLTR2 receptors were medium in cancer tissue but low in normal epithelium of cancer patients and control basal stratum. Protein expression of LTB4R2 and CYSLTR2 was found to be significantly increased in cancer tissue compared to normal mucosa of cancer group and control (*p* < 0.05). With respect to CYSLTR1 expression, cancer tissue displayed a significant up-regulation compared to non-transformed squamous epithelium of cancer patients (*p* < 0.05) but not to control epithelium of dyspeptic patients. Protein expression of LTB4R2, CYSLTR1 and CYSLTR2 in basal stratum of non-transformed epithelium of cancer patients did not differ from the corresponding layer in control.

**Table 2:** Immunohistochemical expression of leukotriene receptors

Immunoreactive score (IRS)				
	LTB4R	LTB4R2	CYSLTR1	CYSLTR2
CSE median (range)	4 (0 - 8)	4 (4 - 6)	4 (4 - 8)	4 (-)
NTSE median (range)	8 (4 - 8)	4 (0 - 4)	4 (0 - 8)	4 (-)
ESCC median (range)	8 (4 - 12)	8 (4 - 12)	8 (4 - 12)	8 (4 - 12)
NTSE/CSE, p-value	**0.034**	0.588	1	1
ESCC/CSE, p-value	**0.0016**	**0.016**	0.252	**0.016**
ESCC/NTSE, p-value	1	**0.0059**	**0.047**	**0.0234**

IRS of all receptors are shown in the upper part of the table. Medians of IRS and corresponding range of staining intensities are shown for each group. In the lower part of the table comparisons between the different histological groups are shown. For statistical analysis, Wilcoxon signed-rank test was applied on groups of cancer patient whereas Mann-Whitney *U* test was used for inter-individual comparisons. All p-values were multiplied by k = 12 (Bonferroni correction) and considered significant when <0.05. Significant changes are displayed in bold letters. *ESCC* esophageal squamous cell cancer, *NTSE* non-transformed squamous epithelium of cancer patients, *CSE* control squamous epithelium; *p* < 0.05 (Wilcoxon signed-rank test, Mann-Whitney *U* test, Bonferroni correction k = 12)

Immune cells infiltrating cancerous and non-cancerous tissue also presented a cytosolic and occasionally membranous leukotriene receptor expression for the various receptors.

### Expression of *LTB4R/LTB4R2* and *CYSLTR1/CYSLTR2* mRNA in cancer tissue, non-transformed epithelium of cancer patients and control

**Fig. 3:**
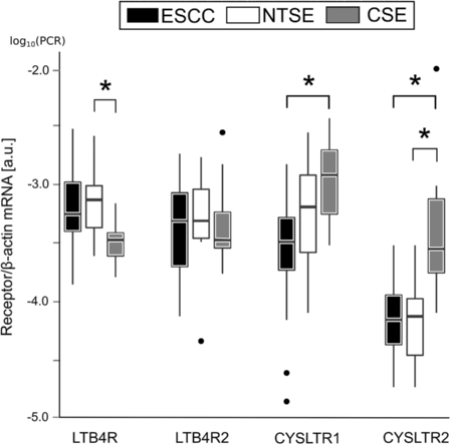
mRNA expression of the leukotriene receptors in patients with esophageal squamous cell cancer (cancer and non-transformed mucosa) and in control. Logarithmic values were used to calculate the representation of each box plot. Median values (line within the box), and 25 % and 75 % quartiles (*upper* and *lower* box border) are shown. Brackets represent significant 2-group comparisons. Dots indicate values below or above the whiskers. a.u. = arbitrary units; ESCC = esophageal squamous cell cancer; NTSE = non-transformed squamous epithelium of cancer patients; CSE = control squamous epithelium; * = *p* < 0.05 (Wilcoxon-Test, Mann-Whitney *U*-Test)

Raw data on mRNA expression of leukotriene receptors are provided in the Additional file 2. Figure 3 shows the mRNA transcript level of *LTB4R/LTB4R2* and *CYSLTR1/CYSLTR2* in the 3 groups. The *LTB4R* mRNA showed an increase of the transcript in cancer tissue (1.69-fold) and non-transformed esophageal epithelium of cancer patients (2.24-fold) compared to control. The increase of *LTB4R* mRNA in normal mucosa of patients with cancer compared to control was statistically significant (*p* < 0.05). No significant differences were found between ESCC and non-transformed mucosa in cancer patients. The *LTB4R2* transcript showed no statistically significant difference between cancer tissue and the esophageal squamous epithelium (patients with ESCC and control). In cancer tissue, *CYSLTR1* showed a decrease to 0.26-fold in mRNA levels compared to control with statistical significance (*p* < 0.05). Between cancers and non-transformed mucosa of cancer patients, no differences were statistically detectable. The expression of *CYSLTR2* mRNA was reduced in cancer tissue to 0.23-fold and in normal epithelium of cancer patients to 0.25-fold compared to control. This reached statistical significance (*p* < 0.05).

### Expression of leukotriene receptors and tumor stage

The clinical classification of tumor stages was performed according to UICC [21]. As the numbers of patients in stages I-III were small, stage I, II and III were grouped together and compared with stage IV. There was no observed difference in the expression of the receptors LTB4R, LTB4R2, CYSLTR1 and CYSLTR2 between the groups (Mann-Whitney *U* test).

## Discussion

Herein we report for the first time the expression of leukotriene receptors in ESCC as well as in esophageal squamous epithelium of patients with and without esophageal neoplasia. The role of leukotriene receptors in immune cells is established [22], but their role in the esophageal mucosa has not been investigated so far, and thus future studies focusing on the physiological role of leukotriene receptors in the esophageal mucosa are warranted.

In the present study we report an up-regulation of LTB4R protein in ESCC compared to control. Similarly, a trend toward an up-regulation of *LTB4R* transcripts in ESCC compared to control was observed. An up-regulation of LTB4R protein has been reported in gastric and pancreatic cancer as well [15, 23]. However, combining the LTB4R antagonist LY293111 to gemcitabine did not add any benefit in terms of survival to chemotherapy-naïve patients with advanced pancreatic carcinoma [24]. Whether the use of a LTB4R antagonist might be an option for treating ESCC has not been studied yet. LTB4R expression was observed to be increased in the proliferative zone of gastric epithelium [25]. Moreover, in a placebo controlled trial on human volunteers, the oral LTB4-receptor antagonist VML295 diminished the proliferative epidermal activity after traumatically induced skin lesions [26]. LTB4R showed an up-regulation in proliferative areas of esophageal mucosa as well, suggesting a role of this receptor for the proliferation of esophageal squamous epithelium. Interestingly, an increased expression of LTB4R was observed also in non-transformed esophageal mucosa of cancer patients compared to control. This phenomenon suggests a potential role of LTB4R in early steps of esophageal carcinogenesis.

LTB4R2 protein expression is also up-regulated in ESCC whereas the specific transcripts did not show any difference between cancerous and non-cancerous epithelia. Previous studies have shown an increased LTB4R2 receptor expression in different epithelial cancers, suggesting a role of this receptor in cancer spread [27, 28]. In a recent study on an orthotopic breast cancer model, increasing the expression of the LTB4R2/LTB4/12(S)-HETE receptor system by lipopolysaccharide stimulation enhanced invasion of breast cancer cells, whereas its selective inhibition turned out in a reduced number of metastatic nodules [29]. Whether pharmacological inhibition of LTB4R2 might offer new options for treatment of ESCC has still to be determined.

LTB4R2 has not been described in normal esophageal mucosa before. The expression of LTB4R2 has been reported in different tissues and apart from its role in inflammatory cells LTB4R2 function needs to be clarified [30]. In a mice model, LTB4R2 was found to be expressed in colon cryptic cells and had a protective role against dextran sodium sulfate (DSS)-induced colitis, possibly by enhancing barrier function in epithelial cells of the colon [31]. A protective function of the LTB4R2 is to assume for the esophageal epithelium as well.

CYSLTR1 and CYSLTR2 proteins are up-regulated in ESCC, while the specific transcripts show a down-regulation compared to the esophageal mucosa of dyspeptic patients. An increased expression of CYSLTR1 receptor protein was described in gastrointestinal and urological malignancies before [15, 32], whereas a loss of CYSLTR2 protein expression was shown in colon cancer cells compared to non-malignant intestinal cells [33]. According to functional studies, CYSLTR1 mediates preferentially pro-carcinogenic effects, whereas CYSLTR2 has been associated with anti-tumor mechanisms. In a recent study on colon cancer cells, CYSLTR1 signaling induced β-catenin translocation and the activation of β-catenin target genes, resulting in increased proliferation and migration of colon cancer cells [34]. Furthermore, CYSLTR1 expression was correlated with enhanced levels of anti-apoptotic proteins in colon cancer and *CYSLTR1* transfection resulted in prolonged survival of Caco-2 cancer cells in vitro [35]. With respect to the CYSLTR2 function, a study on 329 colorectal cancers showed a more favorable prognosis for patients with high nuclear CYSLTR2 staining in combination with low nuclear CYSLTR1 receptor. Statistical analysis found high CYSLTR2 expression associated with a decreased risk of death [36]. In another study, aggressive CYSLTR2 negative breast cancer cells (MDA-MB-231) exhibited a decrease in migratory capacity after *CYSLTR2* transfection which is associated with a reduction in metastatic potential [37]. Whether CYSLTR1 and CYSLTR2 might promote carcinogenesis and spread of ESCC needs to be addressed in future studies. The observed discordance between CYSLTR1, CYSLTR2 protein expression and gene transcription can be explained by the presence of leukotriene receptor positive inflammatory cells infiltrating transformed and non-transformed tissue of cancer patients. An increase of β-actin in these leukocytes might have biased RT-PCR results in these patients mocking a total lower cysteinyl leukotriene receptor transcription. Furthermore, within the esophageal mucosa, spinosal and superficial cell layers constitute the main part of cellular mass. In the immunohistochemical analysis, the comparison was focused on the protein expression of cancer tissue and basal stratum only whereas in the RT-PCR all esophageal layers were analyzed. Thus, methodological aspects explain the differences between protein and mRNA expression pattern of the different leukotriene receptors.

The expression of CYSLTR1 and CYSLTR2 receptors was observed in non-transformed esophageal tissue as well. Similarly, non-transformed esophageal mucosa of cancer patients showed a significant reduction in *CYSLTR2* mRNA transcription compared to the esophageal mucosa of dyspeptic control patients. Synthesis of cysteinyl leukotriene by the esophageal mucosa has been demonstrated, suggesting a physiological role of these receptors within the esophageal mucosa [38]. Further studies to define the role of cysteinyl leukotriene receptors in esophageal squamous epithelium are warranted.

The relatively small groups of patients and controls represent the major limitation of our study. This resulted in limited statistical power for some of the subgroups analysis and prevented stratified analysis by sex and some other risk modifiers. Moreover, the small number of recruited patients may be responsible for the lack of correlation between leukotriene receptor expression and ESCC stage (I-III versus IV). Larger studies may provide clearer results.

Furthermore, all inflammatory cells revealed a constant expression of leukotriene receptors. Cancerous and non-cancerous tissue of patients often showed a varying degree of inflammatory cell infiltration which may have influenced results of RT-PCR.

In addition, biopsies were not checked by microdissection for content of cancerous and non-cancerous tissue. Thus, the presence of non-transformed epithelium in cancer samples cannot be excluded and may have influenced results of mRNA analysis.

Another limitation is the observational character of this study. Although we explored leukotriene receptor expression in ESCC, the functional relevance of this finding remains to be determined. Functional research was not planned beforehand. Future in vitro analysis using siRNA knock-down techniques could clearly address this issue elucidating the role of leukotriene receptors in ESCC.

## Conclusions

In the present study we report a deregulated expression of leukotriene receptors in esophageal squamous cell carcinoma. Furthermore, our results suggest a possible up-regulation of *LTB4R* and down-regulation of *CYSLTR2* gene expression also in the adjacent non-transformed squamous epithelium of the esophagus. Our data may implicate a potential role of these receptors in the early steps of esophageal carcinogenesis. Further studies with a larger number of patients and controls are warranted to validate our findings and to determine clinical implications for therapeutic and prevention purposes.

## Abbreviations

cDNA, complementary desoxyribonucleic acid; CSE, control surface epithelium; CYSLTR1/2, cysteinyl leukotriene receptor 1/2; dNTP, desoxyribonucleic triphosphate; GI, gastrointestinal tract; IHC, immunohistochemistry; IRS, immunoreactive score; LTB4R, leukotriene B4 receptor 1; LTB4R2, leukotriene B4 receptor 2; mRNA, messenger ribonucleic acid; NTSE, non-transformed surface epithelium; PP, percentage of positive cells; PPI, proton pump inhibitor; qRT-PCR, quantitative reverse transcription-polymerase chain reaction; SI, staining intensity; UICC, Union Contre le Cancer

## Additional files

Additional file 1: Study questionnaire. (DOC 21 kb) Additional file 2: Raw data of immunohistochemical scores and polymerase chain reactions. (XLS 10 kb)
