# Supplementary material for: Leukotriene receptor expression in esophageal squamous cell cancer and non-transformed esophageal epithelium: a matched case control study
Source: BMC Gastroenterol. 2016 Jul 30;16:85. doi: 10.1186/s12876-016-0499-z (PMC4967508; doi:10.1186/s12876-016-0499-z)
Supplement: Additional file 1: — Study questionnaire. (DOC 21 kb) [file 12876_2016_499_MOESM1_ESM.doc]

### Study Questionnaire Pat. No.:

**Patient** adhesive label (name, surname, date of birth, department)

**Date of endoscopic examination**______________

Indication for endoscopy/gastrointestinal medical history: ____________________________________________________________________________

**Dominating symptoms responsible for consulting a physician**:

## Loss of weight Dysphagia Heartburn Odynophagia

Since__________________________

Presence of a further malignant tumor _____________________________________________

Further relevant diagnoses: ____________________________________________________________________________

## **Medication**

|  | name of medication | dose | since | until | remarks |
| --- | --- | --- | --- | --- | --- |
| PPI |  |  |  |  |  |

Further medications: ____________________________________________________________________________

**Height:** ____ cm **Weight**:____kg

**Smoker**: yes N° of cigarettes_______  **never**

**former smoker** **since**______ ; smoked for ______years N° _____cigarettes/day

**Alcohol:** never < 10 g/day < 20 g/day < 40 g/day > 40 g/day

**abstinent** **since** ______ ; been active alcohol drinker for ______years

**Alcohol** (if yes): Wine Beer Hard liquor

(10g Alcohol = ca .200 ml Beer ca. 100 ml Wine 20-30 ml 30-50% Alcohol)

Remarks:
